# Clinical Characteristics and Sex Hormone Levels in 323 Cases of Hypospadias With or Without Associated Malformations

**DOI:** 10.3390/children13070867

**Published:** 2026-06-29

**Authors:** Haosen Shen, Ling Li, Ying Qiu, Kexin Zhang, Jiaxin Cheng, Shuangshuang Yang, Xianghui Xie, Qin Zhang

**Affiliations:** 1 Capital Institute of Pediatrics, Chinese Academy of Medical Sciences & Peking Union Medical College, Beijing 100730, China; 2 Department of Urology, Capital Center for Children’s Health, Capital Medical University, Capital Institute of Pedaitrics, Beijing 100020, China; 3 Beijing Municipal Key Laboratory of Child Development and Nutriomics, Capital Center for Children’s Health, Capital Medical University, Capital Institute of Pediatrics, Beijing 100020, China; 4 Department of Urology, Beijing Friendship Hospital, Capital Medical University, Beijing 100050, China

**Keywords:** hypospadias, associated anomalies, epidemiology, sex hormones

## Abstract

**Highlights:**

**What are the main findings?**

• Hypospadias with urogenital-related anomalies showed distinct hormonal characteristics compared with isolated hypospadias.
• After propensity score matching, patients with urogenital-related anomalies showed modestly higher FSH and PRL levels, whereas no significant hormonal differences were observed in those with non-urogenital anomalies.

**What are the implications of the main findings?**

• Hypospadias with urogenital anomalies may represent a clinically distinct subgroup.
• These patients need further endocrine follow-up.

**Abstract:**

**Objectives**: Hypospadias is one of the most common congenital malformations of the male genitourinary system and is frequently accompanied by other congenital anomalies. Whether patients with associated malformations exhibit distinct endocrine characteristics remains unclear. **Methods**: A retrospective analysis was performed on 323 hypospadias patients who underwent surgery at Capital Medical University Affiliated Capital Children’s Medical Center between January 2020 and September 2024. Data on surgical age, clinical classification, associated malformations, and sex hormone levels were collected. Propensity score matching was used for between-group comparisons. **Results**: Associated malformations were present in 68 of 323 patients (21.05%), most commonly involving the urogenital system (75.00%). Cryptorchidism was the most common associated anomaly. The surgical age was significantly later in patients with associated malformations (median 3.34 years) compared to those with isolated hypospadias (2.42 years). Following matching, patients with urogenital-related anomalies had significantly elevated FSH and PRL levels (both *p* < 0.05) compared with isolated hypospadias, whereas no significant hormonal differences were found in those with non-urogenital anomalies. **Conclusions**: Children with hypospadias and associated urogenital anomalies exhibited subtle differences in endocrine profiles compared with isolated hypospadias. These findings suggest that hypospadias with urogenital anomalies may represent a clinically distinct subgroup and support the need for further prospective studies and long-term endocrine follow-up.

## 1. Introduction

Hypospadias is one of the most common congenital malformations of the male genitourinary system. It is mainly characterized by an abnormal location of the urethral opening and is often accompanied by ventral penile curvature and anomalous foreskin distribution [1,2]. The etiology of hypospadias remains incompletely understood. Current evidence suggests that it results from multiple interacting mechanisms, including endocrine disruption, genetic mutations and environmental exposures [3]. Inadequate androgen stimulation is considered a primary etiological factor in the development of hypospadias [4]. The incidence of hypospadias varies between different countries and regions but has exhibited a generally increasing trend over time [5,6]. Approximately 15–25% of hypospadias cases are associated with additional congenital anomalies involving other systems [5]. However, large-scale epidemiological studies and detailed clinical characterizations of hypospadias, particularly in patients with associated malformations, remain scarce in China. Therefore, this study retrospectively analyzed the clinical data of 323 patients with hypospadias who underwent surgery at the Capital Medical University Affiliated Capital Children’s Medical Center between January 2020 and September 2024. The study aimed to investigate the clinical characteristics such as age distribution, clinical classification, associated anomalies, and sex hormone levels, in order to provide scientific basis for the etiological research and individualized clinical management of hypospadias.

## 2. Materials and Methods

### 2.1. Participants

A total of 323 patients with hypospadias who underwent surgery at the Department of Urology, Capital Medical University Affiliated Capital Children’s Medical Center, from January 2020 to September 2024 were enrolled in this study. Exclusion criteria were as follows: (1) patients with unclear classification; (2) patients with isolated penile curvature; (3) patients with chromosomal abnormalities; (4) patients with incomplete clinical documentation. The study was approved by the Institutional Ethics Committee (Approval Number: SHERLL2023038), with informed consent waived due to the retrospective nature of the study.

### 2.2. Data Collection and Clinical Classification

The following clinical data were collected for all patients with hypospadias: age at surgery, clinical classification, height, weight, and the presence of associated malformations. Based on the location of the urethral meatus, hypospadias was classified into three types [7]: (1) distal type (including glanular, coronal, and subcoronal variants); (2) midshaft type (penile); and (3) proximal type (including penoscrotal, scrotal, and perineal variants).

### 2.3. Sex Hormone Detection

All study participants provided fasting venous blood samples in the morning between 7:00 and 9:00 a.m. to minimize diurnal and physiological variations. We measured the serum levels of sex hormones, including follicle-stimulating hormone (FSH), luteinizing hormone (LH), prolactin (PRL), and progesterone (P4), using an automated chemiluminescence immunoassay analyzer (Roche Cobas e601, manufactured by Roche Diagnostics, Switzerland).

### 2.4. Statistical Analyses

Qualitative variables were presented as frequencies and percentages, while quantitative variables were expressed as mean ± standard deviation (SD) for normally distributed data or median with interquartile range (IQR) for non-normally distributed data. Comparative analyses were conducted using Student’s *t*-test for parametric data and the Mann–Whitney U test for non-parametric data. A two-sided *p* < 0.05 was considered statistically significant. All data analyses were performed using the SPSS software version 27.0 (IBM, Armonk, NY, USA).

## 3. Results

### 3.1. General Characteristics and Clinical Classification

A total of 323 patients with hypospadias were included, with ages ranging from 0.50 to 12.83 years and a median age of 2.50 years (IQR: 1.58–4.17). Among them, 255 cases (78.95%) were classified as isolated hypospadias (median age: 2.42 years, IQR: 1.50–3.83), while 68 cases (21.05%) presented with associated malformations (median age: 3.34 years, IQR: 2.00–5.00).

Based on the anatomical location of the urethral meatus, the total group included 73 cases (22.60%) of distal hypospadias, 170 cases (52.63%) of midshaft type, and 80 cases (24.77%) of proximal type. In the isolated hypospadias group (*n* = 255), the distribution was as follows: distal type in 62 cases (24.31%), midshaft type in 140 cases (54.90%), and proximal type in 53 cases (20.78%). In the group with associated malformations (*n* = 68), distal type accounted for 11 cases (16.18%), midshaft type for 30 cases (44.12%), and proximal type for 27 cases (39.71%).

These findings indicate that patients with associated malformations had a higher proportion of proximal hypospadias, suggesting a more complex clinical presentation.

### 3.2. Hypospadias with Associated Malformations

Among the 68 patients with hypospadias and associated malformations, the distribution of affected systems was as follows: cardiovascular system malformations in 15 cases (22.06%), urogenital system malformations in 51 cases (75.00%), musculoskeletal system malformations in 5 cases (7.35%), digestive system malformations in 3 cases (4.41%), and other malformations in 12 cases (17.65%). Regarding the number of systems involved, 49 cases (72.06%) presented with malformations in a single system, 16 cases (23.53%) in two systems, and 3 cases (4.41%) in three or more systems. According to the involvement of the urogenital system, patients were divided into two subgroups: the urogenital-related anomaly group (*n* = 56), comprising patients with anomalies of the urogenital tract, external genitalia, or closely related structures such as inguinal hernia, and the non-urogenital anomaly group, including malformations of the cardiovascular and musculoskeletal systems, among others (*n* = 12) (Table 1).

### 3.3. Sex Hormone Levels in Patients with Hypospadias

**Figure 1:**
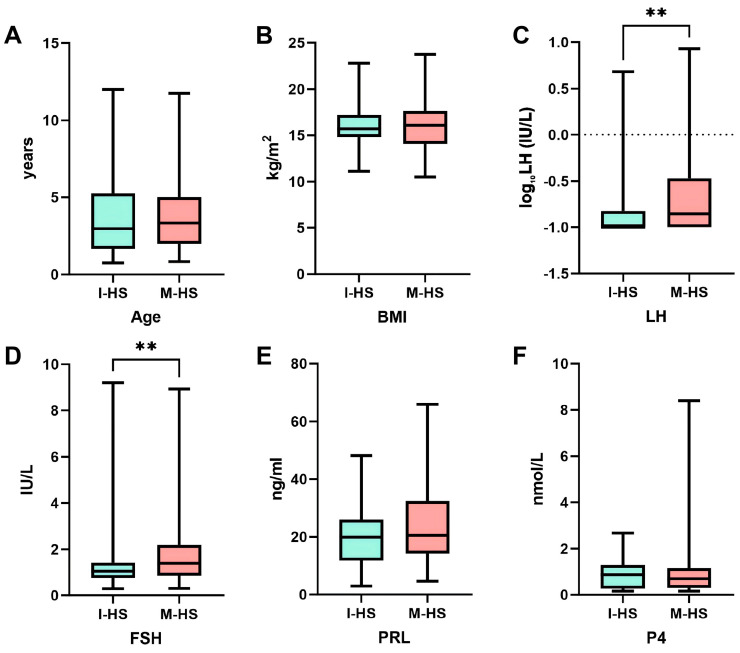
Box plot comparison of clinical and hormonal parameters between patients with isolated hypospadias (I−HS) and those with hypospadias associated with malformations (M−HS): (**A**) age (years), (**B**) BMI (kg/m^2^), (**C**) log_10_LH (IU/L), (**D**) FSH (IU/L), (**E**) PRL (ng/mL), and (**F**) P4 (nmol/L). ** *p* < 0.01. I−HS, isolated hypospadias; M−HS, hypospadias with associated malformations; BMI, body mass index; LH, luteinizing hormone (log_10_−transformed for visualization, statistical comparisons were performed on original values); FSH, follicle-stimulating hormone; PRL, prolactin; P4, progesterone.

To compare sex hormone levels between patients with isolated hypospadias and those with associated malformations, propensity score matching (PSM) was performed at a 1:1 ratio based on age and BMI, with a caliper width of 0.02. After matching, patients in the associated malformations group exhibited significantly higher levels of LH and FSH compared to those in the isolated hypospadias group (*p* < 0.05, Table 2 and Figure 1). These findings prompted further subgroup analyses to determine whether the observed hormonal differences were primarily associated with urogenital-related anomalies or with associated malformations in general.

**Figure 2:**
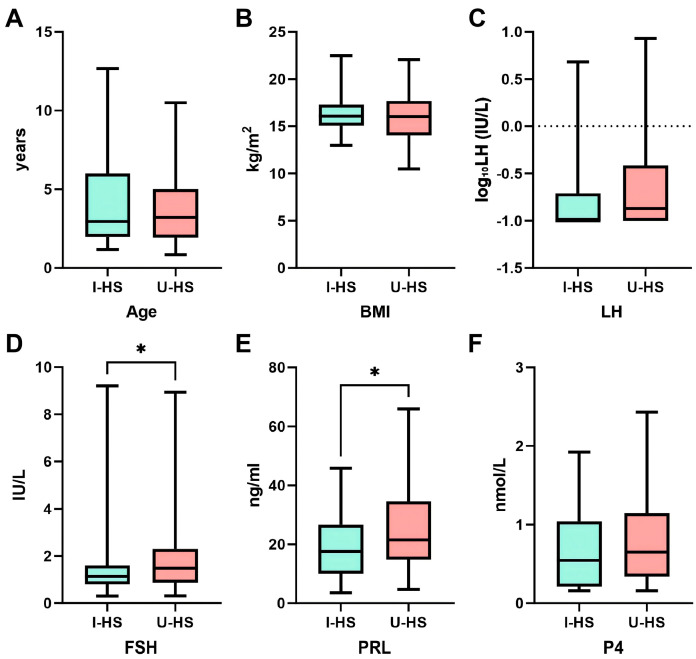
Box plot comparison of clinical and hormonal parameters between patients with isolated hypospadias (I−HS) and those with hypospadias with urogenital-related anomalies (U−HS): (**A**) age (years), (**B**) BMI (kg/m^2^), (**C**) log_10_LH (IU/L), (**D**) FSH (IU/L), (**E**) PRL (ng/mL), and (**F**) P4 (nmol/L). * *p* < 0.05. I−HS, isolated hypospadias; U−HS, Hypospadias with urogenital-related anomalies; BMI, body mass index; LH, luteinizing hormone (log_10_−transformed for visualization, statistical comparisons were performed on original values); FSH, follicle-stimulating hormone; PRL, prolactin; P4, progesterone.

**Figure 3:**
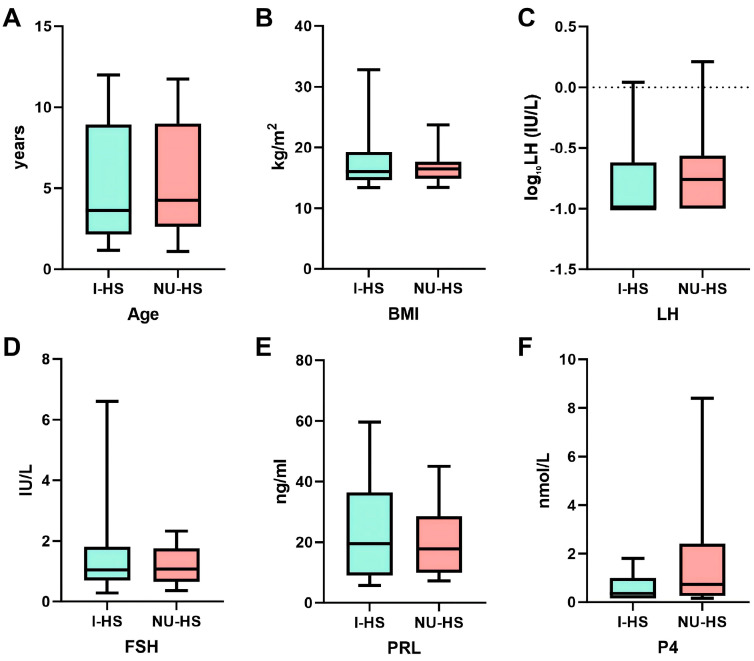
Box plot comparison of clinical and hormonal parameters between patients with isolated hypospadias (I−HS) and those with hypospadias with non-urogenital anomalies (NU−HS): (**A**) age (years), (**B**) BMI (kg/m^2^), (**C**) log_10_LH (IU/L), (**D**) FSH (IU/L), (**E**) PRL (ng/mL), and (**F**) P4 (nmol/L). I−HS, isolated hypospadias; NU−HS, Hypospadias with non-urogenital anomalies; BMI, body mass index; LH, luteinizing hormone (log_10_−transformed for visualization, statistical comparisons were performed on original values); FSH, follicle-stimulating hormone; PRL, prolactin; P4, progesterone.

Sex hormone levels were compared between patients with isolated hypospadias and those with urogenital-related anomalies, as well as between patients with isolated hypospadias and those with non-urogenital anomalies. Propensity score matching (PSM) was performed separately for each comparison at a 1:1 ratio based on age and BMI, with a caliper width of 0.02. After matching, patients in the urogenital-related anomaly group exhibited significantly higher levels of FSH and PRL compared to those in the isolated hypospadias group (*p* < 0.05, Table 3 and Figure 2). In contrast, no significant differences in sex hormone levels were observed between patients with non-urogenital anomalies and those with isolated hypospadias (*p* > 0.05, Table 4 and Figure 3).

The observed hormonal differences were mainly present in patients with urogenital-related anomalies, suggesting that endocrine alterations may be more closely associated with abnormalities involving the male reproductive system rather than with associated malformations overall.

## 4. Discussion

### 4.1. Clinical Characteristics of Hypospadias

A common congenital malformation of the male genitourinary system, the etiology and pathogenesis of hypospadias remain incompletely understood, and large-scale epidemiological data in China are still lacking. In this study, we retrospectively analyzed the clinical data of 323 patients with hypospadias who underwent surgery at our hospital over the past five years, including age distribution, clinical classification, and associated malformations, aiming to systematically characterize its clinical features and provide indirect evidence for understanding the epidemiology of this condition in China.

This study indicates that the overall median age at which patients with hypospadias underwent surgery was 2.50 years (IQR: 1.58–4.17). Patients with isolated hypospadias underwent surgery at a median age of 2.42 years (IQR: 1.50–3.83), whereas those with associated malformations experienced a significant delay to 3.34 years (IQR: 2.00–5.00). This delay may be due to these patients needing to prioritize managing more severe associated anomalies (e.g., congenital heart disease or anorectal malformations) and undergoing comprehensive endocrine and genetic evaluations during early life [8]. Moreover, because hypospadias repair is an elective reconstructive procedure [9], it is usually deferred until other systemic abnormalities have been addressed and the child’s general health has stabilized.

The most common clinical subtype was midshaft (52.47%, 170/323), followed by proximal (24.69%, 80/323) and distal (22.53%, 73/323) types. This distribution pattern differs from the predominantly distal type reported in international studies [10,11], but is consistent with findings from several domestic investigations [12,13]. This disparity could be explained by the milder clinical symptoms, fewer complications and limited long-term impact on reproductive function of distal hypospadias. As a result, parents may be less motivated to have surgery, which could lead to their underrepresentation in surgical cohorts.

Of the 323 patients with hypospadias, 68 (21.05%) presented with additional malformations, which is consistent with previous reports [6]. Urogenital system anomalies were the most prevalent (75.00%, 51/68), primarily involving cryptorchidism and hydrocele. This finding aligns with the results of similar studies [12]. Additionally, 22.06% of patients presented with cardiovascular system abnormalities, confirming the established association between hypospadias and congenital heart disease [14,15,16]. The majority of affected children had anomalies confined to a single system, while involvement of three or more systems was relatively uncommon.

### 4.2. Sex Hormone Levels in Patients with Hypospadias and Associated Malformations

Hypospadias is a congenital malformation resulting from the interaction of endocrine, genetic, and environmental factors. Endocrine regulation plays a particularly critical role in the differentiation of external genitalia during embryonic development [17,18]. The measurement of sex hormone levels in affected children assesses the capacity and functional reserve of testicular androgen synthesis [19]. This provides a theoretical foundation for deciding when surgical repair should be performed and whether preoperative hormone therapy is necessary.

This study compared sex hormone levels among patients with different types of hypospadias. Patients with associated anomalies exhibited overall higher levels of LH and FSH compared to those with isolated hypospadias. Further subgroup analysis revealed that patients with urogenital-related anomalies had significantly elevated FSH and PRL levels, while those with non-urogenital anomalies showed no statistically significant differences in sex hormone levels compared to the isolated group.

These findings suggest that hypospadias with urogenital-related anomalies may involve broader developmental or endocrine alterations beyond localized urethral maldevelopment alone. However, because the study was conducted during childhood, when the hypothalamic–pituitary–gonadal axis is physiologically relatively quiescent, and because direct measurements of testosterone and other gonadal function markers were unavailable, the biological and clinical significance of these hormonal differences remains uncertain. Therefore, the observed endocrine changes should be interpreted cautiously and regarded as preliminary, hypothesis-generating findings rather than evidence of definitive endocrine dysfunction. Further prospective studies incorporating longitudinal endocrine assessment, pubertal evaluation, and long-term follow-up are needed to clarify the relevance of these findings. Notably, studies have demonstrated impaired spermatogenesis and reduced offspring birth rates in adult men with hypospadias [20,21], highlighting the need for clinicians to monitor testicular reserve function and relevant endocrine markers.

By comparison, hypospadias patients with non-urogenital anomalies may have shared developmental abnormalities affecting multiple embryonic systems, without increased risk of HPG axis disruption. Therefore, clinical management should prioritize addressing other severe anomalies, and routine comprehensive endocrine screening may not be necessary. These patients generally exhibit a more favorable endocrine profile, but long-term follow-up is warranted.

This study did not include analysis of testosterone and estrogen, because after the mini-pubertal period, the HPG axis is physiologically suppressed during childhood, and basal levels of both gonadotrophins and sex steroids are low [22]. In our retrospective cohort, the vast majority of testosterone and estrogen values were below the laboratory’s lower limit of detection, precluding valid quantitative comparisons. Thus, the childhood period is not suitable for diagnosing HPG axis disorders in individual patients. The primary objective of our hormone measurements was to compare group-level distributions between phenotypes (isolated hypospadias vs. hypospadias with associated malformations), not to make individual clinical diagnoses. Progesterone was included in our analysis because it was part of the routine sex hormone panel in our hospital’s medical record system, given the retrospective nature of this study. As a common precursor of androgens and adrenal corticosteroids, progesterone levels provide auxiliary assessment of the steroid hormone synthesis pathway. No significant differences in progesterone levels were observed between groups, suggesting that progesterone metabolism pathways may not be significantly disturbed in children with hypospadias.

This study has several limitations. First, as a single-center retrospective study, the representativeness of the sample is limited. Data were derived from the hospital’s electronic medical record system, which has inherent limitations, including incomplete information recording and inconsistent laboratory indicators. Second, the lack of data on potential confounding factors, such as maternal intrauterine endocrine exposure and fetal birth conditions, may affect the interpretation of our findings. Third, genetic test results were not available for analysis; future studies should incorporate genetic data to provide deeper insights. Fourth, the study cohort spanned a broad age range (0.50–12.83 years). We did not assess Tanner staging or account for the potential impact of mini-puberty on hormone levels; thus, we cannot exclude the possibility that some patients were still within the mini-puberty window or had already entered early puberty. Although propensity score matching was used to balance age between comparison groups, it cannot fully eliminate physiological hormone fluctuations associated with mini-puberty or pubertal onset. Finally, hormone levels were measured from a single preoperative blood sample, lacking long-term dynamic follow-up. Prospective studies are warranted to validate these findings.

## 5. Conclusions

In this retrospective study, children with hypospadias accompanied by urogenital-related anomalies exhibited modest differences in serum FSH and PRL levels compared with those with isolated hypospadias, whereas no significant hormonal differences were observed in patients with non-urogenital anomalies. These findings suggest that hypospadias with urogenital-related anomalies may represent a clinically distinct subgroup rather than simply a localized urethral malformation.

Given the retrospective design and the limitations related to childhood endocrine evaluation, the clinical significance of these hormonal differences should be interpreted cautiously. Further prospective studies incorporating longitudinal endocrine assessment and long-term follow-up are warranted to clarify the biological and clinical relevance of these findings.

## Figures and Tables

**Table 1:** Associated malformations in hypospadias patients (*n* = 68).

Malformation	Cases
Cardiovascular system	15
Ventricular septal defect	10
Atrial septal defect	4
Patent ductus arteriosus	2
Patent foramen ovale	6
Urogenital system	51
Cryptorchidism	19
Hydrocele	21
Congenital penoscrotal transposition	12
Micropenis	4
Buried penis	1
Posterior urethral valves	1
Congenital adrenal hyperplasia	1
Musculoskeletal system	5
Digit abnormalities	2
Spinal abnormalities	2
Congenital elevation of scapula (Sprengel deformity)	1
Digestive system	3
Anorectal malformation	3
Other malformations	13
Inguinal hernia	10
Umbilical hernia	1
Congenital diaphragmatic hernia	1

**Table 2:** Comparison of sex hormone levels between patients with isolated hypospadias and those with associated malformations.

	Isolated Hypospadias (*n* = 68)	Hypospadias with Associated Malformations (*n* = 68)	*p*-Value
Age (years)	2.96 (1.67, 5.25)	3.34 (2.00, 5.00)	0.483
BMI (kg/m^2^)	15.71 (14.81, 17.20)	16.10 (14.07, 17.63)	0.694
LH (IU/L)	0.10 (0.10, 0.15)	0.14 (0.10, 0.34)	0.009
FSH (IU/L)	1.06 (0.76, 1.43)	1.39 (0.86, 2.19)	0.007
PRL (ng/mL)	19.92 (11.76, 26.04)	20.53 (14.19, 32.39)	0.174
P4 (nmol/L)	0.87 (0.27, 1.30)	0.70 (0.31, 1.16)	0.674

BMI, Body Mass Index; LH, Luteinizing Hormone; FSH, Follicle-Stimulating Hormone; PRL, Prolactin; P4, Progesterone.

**Table 3:** Comparison of sex hormone levels between patients with isolated hypospadias and those with urogenital-related anomalies.

	Isolated Hypospadias (*n* = 56)	Hypospadias with Urogenital-Related Anomalies (*n* = 56)	*p*-Value
Age (years)	2.96 (2.00, 6.00)	3.21 (1.94, 5.00)	0.825
BMI (kg/m^2^)	16.07 (15.06, 17.28)	16.01 (14.04, 17.70)	0.543
LH (IU/L)	0.10 (0.10, 0.20)	0.14 (0.10, 0.39)	0.051
FSH (IU/L)	1.14 (0.80, 1.60)	1.49 (0.88, 2.30)	0.031
PRL (ng/mL)	17.61 (10.06, 26.59)	21.48 (14.85, 34.51)	0.024
P4 (nmol/L)	0.55 (0.21, 1.04)	0.65 (0.34, 1.15)	0.229

BMI, Body Mass Index; LH, Luteinizing Hormone; FSH, Follicle-Stimulating Hormone; PRL, Prolactin; P4, Progesterone.

**Table 4:** Comparison of sex hormone levels between patients with isolated hypospadias and those with non-urogenital anomalies.

	Isolated Hypospadias (*n* = 12)	Hypospadias with Non-Urogenital Anomalies (*n* = 12)	*p*-Value
Age (years)	5.20 ± 3.73	5.32 ± 3.56	0.935
BMI (kg/m^2^)	15.99 (14.58, 19.24)	16.41 (14.84, 17.63)	0.843
LH (IU/L)	0.10 (0.10, 0.24)	0.18 (0.10, 0.27)	0.291
FSH (IU/L)	1.05 (0.71, 1.81)	1.08 (0.66, 1.76)	0.887
PRL (ng/mL)	24.00 ± 16.97	20.12 ± 11.57	0.519
P4 (nmol/L)	0.37 (0.16, 1.00)	0.74 (0.26, 2.41)	0.219

BMI, Body Mass Index; LH, Luteinizing Hormone; FSH, Follicle-Stimulating Hormone; PRL, Prolactin; P4, Progesterone.

## Data Availability

The data presented in this study are available on request from the corresponding author to ensure patient privacy.

## Abbreviations

The following abbreviations are used in this manuscript:

FSH	Follicle-stimulating hormone
LH	Luteinizing hormones
PRL	Prolactin
P4	Progesterone
IQR	Interquartile Range
HPG	Hypothalamic–Pituitary–Gonadal

## References

[1] Kaefer M. (2021). Hypospadias. Semin. Pediatr. Surg..

[2] van der Horst H.J., de Wall L.L. (2017). Hypospadias, all there is to know. Eur. J. Pediatr..

[3] Kaefer M., Rink R., Misseri R., Winchester P., Proctor C., Ben Maamar M., Beck D., Nilsson E., Skinner M.K. (2023). Role of epigenetics in the etiology of hypospadias through penile foreskin DNA methylation alterations. Sci. Rep..

[4] Holmes N.M., Miller W.L., Baskin L.S. (2004). Lack of defects in androgen production in children with hypospadias. J. Clin. Endocrinol. Metab..

[5] Springer A., van den Heijkant M., Baumann S. (2016). Worldwide prevalence of hypospadias. J. Pediatr. Urol..

[6] Zhiyu C., Yuyang G., Wenli X., Wenyan L., Zhen L., Jiayuan Z., Jun Z., Li D. (2026). Epidemiology of hypospadias in China: A nationwide surveillance-based study, 2010–2020. Andrology.

[7] Braga L.H., Lorenzo A.J., Bagli D.J., Pippi Salle J.L., Caldamone A. (2016). Application of the STROBE statement to the hypospadias literature: Report of the international pediatric urology task force on hypospadias. J. Pediatr. Urol..

[8] Goel A., Goel A. (2024). Optimal timing for plastic surgical procedures for common congenital anomalies: A review. World J. Clin. Pediatr..

[9] Halaseh S.A., Halaseh S., Ashour M. (2022). Hypospadias: A Comprehensive Review Including Its Embryology, Etiology and Surgical Techniques. Cureus.

[10] Rania L., Djalila C.R., Zoubir A., Yacine B., Karima S. (2025). Investigating risk factors for hypospadias: Insights from a study in Eastern Algeria. Arch. Pediatr..

[11] Akay M.A., Yıldız G.E. (2021). Impact of gestational and parental factors and maternal intake of progesterone on the development of hypospadias: A retrospective case-control study. Taiwan. J. Obstet. Gynecol..

[12] Liu Q., Shan Z.C., Liang R.Z., Su Z.L., Zhou X.H. (2019). Clinical features and pathogenic risk factors of hypospadias in children. J. Clin. Ped. Sur..

[13] Xu G.S., Wang A.H., Ni J.X., Zhu Y.H., Zheng Y., Yu L. (2016). Analysis of Epidemiological Characteristics of Hypospadias in Shaanxi Area from 2000 to 2015. Chin. Gen. Pract. J..

[14] Richard M.A., Patel J., Benjamin R.H., Bircan E., Canon S.J., Marengo L.K., Canfield M.A., Agopian A.J., Lupo P.J., Nembhard W.N. (2022). Prevalence and Clustering of Congenital Heart Defects Among Boys With Hypospadias. JAMA Netw. Open.

[15] Ludorf K.L., Benjamin R.H., Navarro Sanchez M.L., McLean S.D., Northrup H., Mitchell L.E., Langlois P.H., Canfield M.A., Scheuerle A.E., Scott D.A. (2021). Patterns of co-occurring birth defects among infants with hypospadias. J. Pediatr. Urol..

[16] Gazdagh G.E., Wang C., McGowan R., Tobias E.S., Ahmed S.F. (2019). Cardiac disorders and structural brain abnormalities are commonly associated with hypospadias in children with neurodevelopmental disorders. Clin. Dysmorphol..

[17] van der Zanden L.F., van Rooij I.A., Feitz W.F., Franke B., Knoers N.V., Roeleveld N. (2012). Aetiology of hypospadias: A systematic review of genes and environment. Hum. Reprod. Update.

[18] Gozar H., Bara Z., Dicu E., Derzsi Z. (2023). Current perspectives in hypospadias research: A scoping review of articles published in 2021 (Review). Exp. Ther. Med..

[19] Tack L.J.W., van der Straaten S., Riedl S., Springer A., Holterhus P.M., Hornig N.C., Kolesinska Z., Niedziela M., Baronio F., Balsamo A. (2022). Growth, puberty and testicular function in boys born small for gestational age with a nonspecific disorder of sex development. Clin. Endocrinol..

[20] Tack L.J.W., Spinoit A.F., Hoebeke P., Riedl S., Springer A., Tonnhofer U., Hiess M., Weninger J., Mahmoud A., Tilleman K. (2022). Endocrine outcome and seminal parameters in young adult men born with hypospadias: A cross-sectional cohort study. EBioMedicine.

[21] Skarin Nordenvall A., Chen Q., Norrby C., Lundholm C., Frisén L., Nordenström A., Almqvist C., Nordenskjöld A. (2020). Fertility in adult men born with hypospadias: A nationwide register-based cohort study on birthrates, the use of assisted reproductive technologies and infertility. Andrology.

[22] Li J.R., Goodman X., Cho J., Holditch-Davis D. (2021). The Variability and Determinants of Testosterone Measurements in Children: A Critical Review. Biol. Res. Nurs..

